# Randomized Trial of Information for Older Women About Cessation of Breast Cancer Screening Invitations

**DOI:** 10.1007/s11606-024-08656-3

**Published:** 2024-02-26

**Authors:** Jenna Smith, Erin Cvejic, Nehmat Houssami, Mara A. Schonberg, Wendy Vincent, Vasi Naganathan, Jesse Jansen, Rachael H. Dodd, Katharine Wallis, Kirsten J. McCaffery

**Affiliations:** 1 Sydney Health Literacy Lab, Sydney School of Public Health, Faculty of Medicine and Health, The University of Sydney, Sydney, NSW Australia; 2 The Daffodil Centre, The University of Sydney, a joint venture with the Cancer Council NSW, Sydney, NSW Australia; 3 Department of Medicine, Beth Israel Deaconess Medical Center, Harvard Medical School, Boston, MA USA; 4 BreastScreen NSW, Sydney Local Health District, Sydney, NSW Australia; 5 Faculty of Medicine and Health, Concord Clinical School, The University of Sydney, Sydney, Australia; 6 Department of Geriatric Medicine, Centre for Education and Research On Ageing, Concord Hospital, Concord, NSW Australia; 7 Faculty of Health, Medicine and Life Sciences, School for Public Health and Primary Care, Maastricht University, Maastricht, Netherlands; 8 General Practice Clinical Unit, Medical School, The University of Queensland, Brisbane, QLD Australia; 9 Edward Ford Building (A27), The University of Sydney, Sydney, NSW Australia

## Abstract

**Background:**

Older women receive no information about why Australia’s breast screening program (BreastScreen) invitations cease after 74 years. We tested how providing older women with the rationale for breast screening cessation impacted informed choice (adequate knowledge; screening attitudes aligned with intention).

**Methods:**

In a three-arm online randomized trial, eligible participants were females aged 70–74 years who had recently participated in breast screening (within 5 years), without personal breast cancer history, recruited through Qualtrics. Participants read a hypothetical scenario in which they received a BreastScreen letter reporting no abnormalities on their mammogram. They were randomized to receive the letter: (1) without any rationale for screening cessation (control); (2) with screening cessation rationale in printed-text form (e.g., downsides of screening outweigh the benefits after age 74); or (3) with screening cessation rationale presented in an animation video form. The primary outcome was informed choice about continuing/stopping breast screening beyond 74 years.

**Results:**

A total of 376 participant responses were analyzed. Compared to controls (*n* = 122), intervention arm participants (text [*n* = 132] or animation [*n* = 122]) were more likely to make an informed choice (control 18.0%; text 32.6%, *p* = .010; animation 40.5%, *p* < .001). Intervention arm participants had more adequate knowledge (control 23.8%; text 59.8%, *p* < .001; animation 68.9%, *p* < .001), lower screening intentions (control 17.2%; text 36.4%, *p* < .001; animation 49.2%, *p* < .001), and fewer positive screening attitudes regarding screening for themselves in the animation arm, but not in the text arm (control 65.6%; text 51.5%, *p* = .023; animation 40.2%, *p* < .001).

**Conclusions:**

Providing information to older women about the rationale for breast cancer screening cessation increased informed decision-making in a hypothetical scenario. This study is an important first step in improving messaging provided by national cancer screening providers direct to older adults. Further research is needed to assess the impact of different elements of the intervention and the impact of providing this information in clinical practice, with more diverse samples.

**Trial Registration:**

ANZCTRN12623000033640.

**Supplementary Information:**

The online version contains supplementary material available at 10.1007/s11606-024-08656-3.

## INTRODUCTION

There is uncertain benefit of screening mammography for older women, as women aged ≥ 74 years have not been included in randomized controlled trials.^1^ At a population level, potential harms become more likely with increasing age, including false positives and anxiety due to additional tests following detection of an abnormality, as well as overdiagnosis (diagnosis of a cancer that would never have caused problems) and overtreatment.^1^ In one Australian modeling study, if screening were extended to 1000 women aged 70–74 years (compared to inviting women up to 69 years), one additional breast cancer death would be averted, eight breast cancers would be overdiagnosed, and 102 additional tests would include 78 additional false positives.^2^ However, a US modeling study indicated that continued screening to 78 or 80 years for women with no comorbidity could have similar benefit-harm ratios as screening women aged 50–74 years at average risk.^3^

United States (US) guidelines recommend individualized breast screening decisions beyond 74 years based on overall health, life expectancy, and personal preference.^3–8^ Screening is not recommended for individual women with limited life expectancy (< 10 years) due to the 10-year lag-time to benefit^9^ and greater chance of harm.^10^ However, over 50% of women aged ≥ 74 years report recent screening in US national survey data,^11,12^ and around 38% of women with limited life expectancy continue breast screening.^13^ In Australia, the nationally funded screening program (BreastScreen) invites women to participate in mammography screening for breast cancer up to 74 years of age through local health districts.^14^ Women aged ≥ 74 years can continue free screening and are encouraged to speak with their primary care clinician (general practitioner [GP]) if they wish.^14^ Screening rates by life expectancy are unknown in Australia. However, since BreastScreen extended invitations to women aged 70–74 years in 2013, annual participation rates in this age group increased from 25.9 to 55.8%, and data suggests 7.6–10% of women continue to be screened beyond 74 years.^15,16^ One reason for this may be the high trust people living in Australia have in the healthcare system.^17^

Older women should be supported to make informed breast screening decisions (i.e., decisions with adequate knowledge of potential benefits/harms, consistent with their values/preferences). However, many have limited knowledge of the potential harms of screening^17^ and believe that they are no longer invited to screen for reasons they consider are ageist and related to government cost-saving or reduced risk of developing cancer.^18,19^ GPs also find it challenging to explain these recommendations.^20^ Multi-level interventions (i.e., patient, clinician, system) are needed to maximize efforts to improve informed decision-making.^21–23^

We conducted a randomized controlled trial to test how different messaging about the rationale for breast screening cessation impacts older women’s decision-making. We included intervention arms via printed-text or animation video. Animations are increasingly used for communicating health information and may result in greater knowledge improvements compared to printed-text materials,^24,25^ particularly for people with low health literacy.^26^

## METHODS

### Study Design

This study was an online, three-arm randomized controlled trial. We followed the Consolidated Standards of Reporting Trials (CONSORT) reporting guideline for randomized clinical trials. Participants were randomly assigned to one of three study arms using a balanced allocation ratio of 1:1:1. The protocol for this trial was registered with the Australian New Zealand Clinical Trials Registry (ANZCTRN12623000033640) and ethical approval was obtained from The University of Sydney’s Human Research Ethics Committee (2022/809).

### Participants

Eligible participants included women aged 70–74 years, living in Australia, who had engaged in mammography screening at least once in the past 5 years. Women who had self-reported a current or previous diagnosis of breast cancer were excluded. This age range was chosen as these women would be approaching the upper age limit for cessation of invitation to the program. They were recruited through Qualtrics, a social research company who partners with many panels to recruit individuals who have already signed up to receive surveys. Upon registration, sample providers verify respondent address, demographic information, and email address. They target eligible participants based on specific parameters including age, gender, and location, but do not guarantee national representation among the invites sent or responses received. They use an incentivization system for participation, for which rewards vary (e.g., cash, gift cards, redeemable points, charitable donations).

### Procedure

Qualtrics’ online survey platform was used to administer the questionnaire and piloting was conducted to test the scenarios and questionnaire. Potential participants were sent an email invitation via Qualtrics with general study information, directed to the study landing webpage where they were able to read the participant information statement, screened for eligibility, and then consented to participation. Participants were presented with a hypothetical scenario in which they imagined they had received a standard BreastScreen letter after a recent mammogram which reported that no breast cancer could be seen. The delivery method of this letter was not specified. This format best represents current Australian BreastScreen practice, reflecting the usual result letters sent to women aged 70–74 years. After reading the letter, participants were randomly allocated to one of three arms:

1. *Control*: standard letter with no additional information.
2. *Intervention—printed-text*: standard letter with additional information on the following page of the survey explaining in bullet points why they will not receive reminders to screen after 74 years.
3. *Intervention—animation video (1 min 51 s long)*: standard letter with additional information in a video on the following page of the survey explaining why they will no longer receive reminders to screen after 74 years. The video included subtitles and narration. A professional animator developed the video based on a script and reference videos provided by the lead researcher.

The messaging in both intervention arms was developed by the research team, including older female health consumers and experts in health communication (JS, RD, JJ, KM), breast cancer screening (NH, MS, WV), geriatric medicine (VN), and general practice (KW). The information explained why women will not receive reminders to screen after 74 years, due to both potential benefits and downsides of screening (overdiagnosis and overtreatment), and because with increasing age and other health issues the chance of experiencing the downsides of screening outweighs the chance of benefit (see Supplementary Material). The interventions were designed to be brief and feasible for delivery at scale. We therefore chose to focus on the harm of overdiagnosis as it is important in older age^27^ and poorly understood by women. In contrast, the harm of false positives is better understood and tolerated by women.^28^ The information also emphasized the decision was up to them but that they could speak with their GP if they were unsure. Iterations were revised based on feedback from the research team, including two consumer experts who advised on acceptability and understandability.

Randomization was conducted automatically using the randomizer function within Qualtrics, which uses the Mersenne Twister pseudorandom number generator. Both participants and researchers were blinded to the allocation sequence until after data collection was completed.

### Outcomes

Immediately after the intervention, participants completed the primary outcome measure of informed choice, which is a binary composite measure (informed/uninformed).^29^ In this hypothetical scenario, participants were considered to have made an informed choice if they had adequate knowledge and reported attitudes regarding screening for themselves that aligned with their intention to stop or continue screening (Box 1). Conceptual knowledge was assessed using 11 true/false questions, adapted from measures used in previous cancer screening decision-making research.^23,29–31^ Adequate knowledge was determined using the median score (i.e., participants had adequate knowledge if their total score was the within-sample median or greater). Personal screening attitudes were assessed using six items scored on a total scale from 6 to 30, with the within-sample median score used to dichotomize into “positive attitudes” and “less than positive attitudes,” as has been done in prior decision aid work.^32^ Screening intention was assessed by a single item with a 5-point scale (1 = Definitely will to 5 = Definitely will not). We decided a priori to dichotomize responses to intending to screen (definitely or probably will) or not intending to screen (unsure or definitely or probably will not), similar to prior decision aid work.^29^ Secondary outcomes included intention to speak to a GP, perceived risk of developing cancer, cancer worry, and emotional response to the letter they received. See Supplementary Table I for further details.

**Table:** **Box 1** Informed Choice Framework.

	Adequate knowledge (scored ≥ within-sample median)*	Positive attitude (score ≥ within-sample median)	Intending to screen (definitely or probably will screen)
Informed choice—adequate knowledge and consistent attitudes + intentions			
Accept screening	✓	✓	✓
Decline screening	✓	-	-
Uninformed choice—inadequate knowledge and/or inconsistent attitudes + intentions			
Accept screening	- / ✓	-	✓
Decline screening	- / ✓	✓	-
Accept screening	-	✓	✓
Decline screening	-	-	-

*Within-sample median threshold was chosen for adequate knowledge because a typical “pass” (or 50%) cut-off would not be appropriate. Participants could, on average, answer 50% of the questions correctly by guessing the answers.

After completing outcome measures, participants answered questions to ascertain sociodemographic and health characteristics, including general health, health literacy,^33^ screening history, personal and family cancer history, and mortality risk.^34–36^ An attention check question and consistency check question were also included to assess the quality of respondent data.

### Statistical Analysis

We conducted a sample size calculation assuming a conservative estimate that 50% of participants in one study arm would make an informed choice. We required 110 participants per arm (330 total) to detect a 20% difference in informed choice between the arms, which would have 80% power (*α* of 5%, two-sided test). Descriptive statistics were calculated for sociodemographic and health characteristics of the sample, and the primary and secondary outcome measures (mean [SD] for continuous variables and frequency and relative frequency for categorical variables). Statistical significance of study arm differences was tested for using chi-squared tests for binary categorical outcomes, ordinal regression models for ordered categorical outcomes, and linear regression models for continuous outcomes. The assumption of parallel lines for ordinal regression models was satisfied. All statistical models included study arm (control, printed-text information, animation video information) as categorical covariates. Where overall differences were identified, planned simple contrasts (e.g., control vs. text intervention, control vs. animation intervention) were conducted. A Bonferroni-adjusted significance threshold of *p* = 0.017 was used for multiple pairwise comparisons.

We conducted sensitivity analyses where participant responses from Western Australia were removed to test whether the outcomes differ, as BreastScreen Western Australia provides older women with information about the upper age of screening programs through their website and final screening letter. This information states, “There is no scientific evidence that for women over 75 participating in a population breast cancer screening program results in more health benefits than harms.”^37^ Other states in Australia do not provide older women with information about why screening invitations stop after 74 years. Sensitivity analysis was also conducted with adjusted thresholds set for primary outcomes (see Supplementary Table IV). SPSS (version 28) was used for all analyses.

## RESULTS

**Figure 1:**
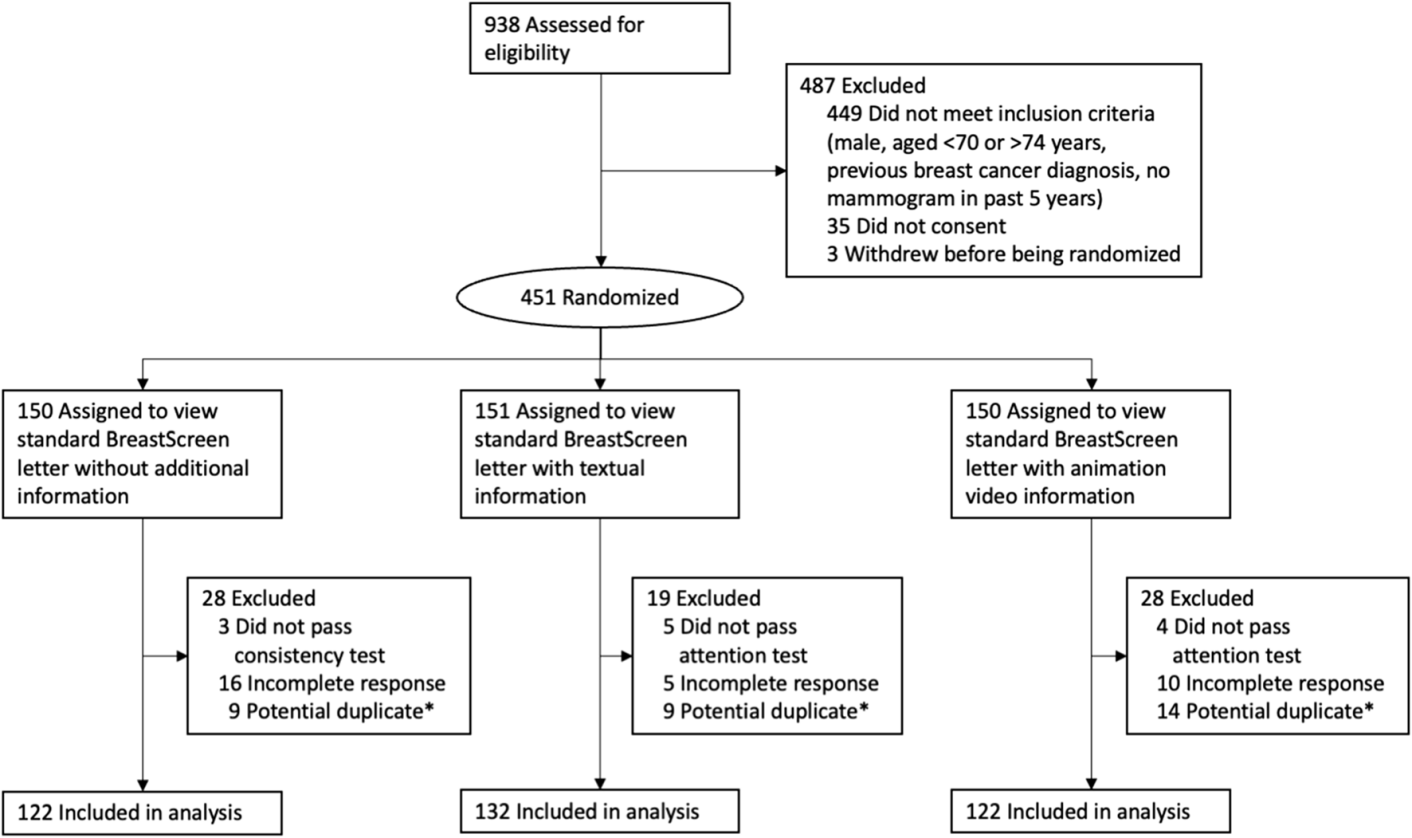
Participant flow diagram. *Potential duplicates were flagged by Qualtrics based on duplicate IP addresses.

A total of 938 respondents accessed the first page of the survey from March 8 to 29, 2023, of whom 451 were randomized and 376 were included in the final analysis (Fig. 1). Characteristics of the participants are presented by study arm in Table 1. Most participants were born in Australia (69.4%) and located in the states of New South Wales, Victoria, or Queensland (80.6%). Half of the participants had an education level of high school or less (51.1%), over half had private health insurance (60.4%), and most reported adequate health literacy (94.1%) using the single-item health literacy screener.^38^

**Table 1:** Characteristics of Participants, Presented by Study Arm and Overall

	Control (*n* = 122)	Text (*n* = 132)	Animation (*n* = 122)	Total (*n* = 376)
Age, M (SD)*	71.76 (1.42)	72.02 (1.45)	71.76 (1.43)	71.85 (1.43)
	N (%)^*^	*N (%)*	*N (%)*	*N (%)*
State				
New South Wales	38 (31.1)	33 (25.0)	30 (24.6)	101 (26.9)
Victoria	33 (27.0)	29 (22.0)	29 (23.8)	91 (24.2)
Queensland	28 (23.0)	43 (32.6)	40 (32.8)	111 (29.5)
South Australia	4 (3.3)	12 (9.1)	13 (10.7)	29 (7.7)
Western Australia	13 (10.7)	12 (9.1)	8 (6.6)	33 (8.8)
Tasmania	4 (3.3)	2 (1.5)	1 (0.8)	7 (1.9)
Australian Capital Territory	2 (1.6)	1 (0.8)	1 (0.8)	4 (1.1)
Northern Territory	0 (0)	0 (0)	0 (0)	0 (0)
Aboriginal or Torres Strait Islander	1 (0.8)	2 (1.5)	2 (1.6)	5 (1.3)
Education				
University degree	19 (15.6)	19 (14.4)	30 (24.6)	68 (18.1)
Diploma, cert, trade	40 (32.8)	52 (39.4)	24 (19.7)	116 (30.9)
High school or less	63 (51.6)	61 (46.2)	68 (55.7)	192 (51.1)
Born in Australia	89 (73.0)	84 (63.6)	88 (72.1)	261 (69.4)
Private health insurance	74 (60.7)	86 (65.2)	67 (54.9)	227 (60.4)
Health literacy (adequate)^+^	114 (93.4)	128 (97.0)	112 (91.8)	354 (94.1)
General health				
Excellent or very good	55 (45.1)	56 (42.4)	39 (32.0)	150 (39.9)
Good	40 (32.8)	54 (40.9)	62 (50.8)	156 (41.5)
Fair or poor	27 (22.1)	22 (16.7)	21 (17.2)	70 (18.6)
Cancer history	17 (13.9)	18 (13.6)	18 (14.8)	53 (14.1)
Breast cancer family history	23 (18.9)	22 (16.7)	25 (20.5)	70 (18.6)
Limited life expectancy (< 10 years)^#^	18 (14.8)	19 (14.4)	14 (11.5)	51 (13.6)
Age of first mammogram*				
< 40 years	31 (25.4)	34 (25.8)	37 (30.3)	102 (27.1)
40–49 years	44 (36.1)	45 (34.1)	43 (35.2)	132 (35.1)
50–59 years	27 (22.1)	27 (20.5)	29 (23.8)	83 (22.1)
60–69 years	5 (4.1)	4 (3.0)	3 (2.5)	12 (3.2)
70–74 years	1 (0.8)	5 (3.8)	3 (2.5)	9 (2.4)
Don’t know	14 (11.5)	17 (12.9)	7 (5.7)	38 (10.1)
Total mammograms^~^				
1	0 (0.0)	2 (1.5)	1 (0.8)	3 (0.8)
2–4	7 (5.7)	8 (6.1)	9 (7.4)	24 (6.4)
5–9	35 (28.7)	35 (26.5)	39 (32.0)	109 (29.0)
10 +	80 (65.6)	87 (65.9)	73 (59.8)	240 (63.8)
Most recent mammogram^~^				
Within past year	52 (42.6)	67 (50.8)	60 (49.2)	179 (47.6)
Between 1 and 2 years ago	53 (43.4)	52 (39.4)	44 (36.1)	149 (39.6)
Between 2 and 5 years ago	17 (13.9)	13 (9.8)	18 (14.8)	48 (12.8)
Mammogram location^~^				
BreastScreen center/van	107 (87.7)	107 (81.1)	95 (77.9)	309 (82.2)
Hospital setting	5 (4.1)	9 (6.8)	11 (9.0)	25 (6.6)
Private screening/medical imaging	10 (8.2)	16 (12.1)	16 (13.1)	42 (11.2)
Called back for follow-up testing	49 (40.2)	33 (25.0)	37 (30.3)	119 (31.6)
Biopsy	26 (21.3)	31 (23.5)	27 (22.1)	84 (22.3)

*All values are numbers and proportions (i.e., *N* (%)), except for age which is reported as means (M) and standard deviation (SD)

^+^Assessed using a single-item self-reported screener that asks whether the respondent needs help when reading written material from a healthcare professional^33^

^#^Assessed using the combined Lee-Schonberg index which is available on the ePrognosis website (https://eprognosis.ucsf.edu/).^36^ Points are accumulated through answers to 15 questions regarding health status and functioning in order to calculate an estimate of risk < 10-year life expectancy^34,35^

^~^Self-reported mammography history

Results for primary and secondary outcomes are presented in-text below, including pairwise comparisons where overall differences were identified between study arms. Table 2 presents results for the primary outcomes by control and intervention arms, including a breakdown of each item contributing to the composite “informed choice” measure. Secondary outcomes by study arm are presented in Table 3.

**Table 2:** Analysis of Primary Outcome. The *p*-Value Provided Is for the Main Effect of Study Arm

Variable	Control (*n* = 122)	Text (*n* = 132)	Animation (*n* = 122)	*p*-value
Knowledge (total score dichotomized using within-sample median [8])				
Adequate knowledge (score ≥ 8/11)	29 (23.8)	79 (59.8)	84 (68.9)	< .001
Total knowledge score (mean, SD, range 0–11)	6.57 (1.27)	7.89 (1.78)	8.44 (1.92)	< .001
Intentions about having breast screening				
Intend to screen	101 (82.8)	84 (63.6)	62 (50.8)	< .001
Do not intend to screen	21 (17.2)	48 (36.4)	60 (49.2)	
Definitely will	68 (55.7)	46 (34.8)	28 (23.0)	…
Probably will	33 (27.0)	38 (28.8)	34 (27.9)	…
Unsure	12 (9.8)	29 (22.0)	32 (26.2)	…
Probably will not	5 (4.1)	14 (10.6)	24 (19.7)	…
Definitely will not	4 (3.3)	5 (3.8)	4 (3.3)	…
Attitudes toward having breast screening^#^				
For you, having breast screening is…				
Beneficial	4.75 (0.71)	4.58 (0.72)	4.45 (0.84)	.008
Harmful (reverse scored)	4.55 (0.93)	4.35 (0.89)	4.29 (0.90)	.062
A good thing	4.77 (0.60)	4.58 (0.79)	4.49 (0.84)	.013
A bad thing (reverse scored)	4.75 (0.75)	4.52 (0.91)	4.44 (0.87)	.015
Worthwhile	4.80 (0.59)	4.62 (0.73)	4.43 (0.88)	< .001
Important	4.83 (0.56)	4.62 (0.74)	4.44 (0.88)	< .001
Mean total attitudes score	28.44 (3.45)	27.27 (4.15)	26.55 (4.29)	.001
Attitudes (dichotomized using within-sample median [30])				< .001
Most positive (score of 30)	80 (65.6)	68 (51.5)	49 (40.2)	
Less than positive (< 30)	42 (34.4)	64 (48.5)	73 (59.8)	
Informed choice* (composite outcome)				
Made an informed choice	22 (18.0)	43 (32.6)	50 (41.0)	< .001

Data are number of participants (%), unless otherwise stated

^#^Six attitude items were rated from strongly disagree (1) to strongly agree (5). Range of possible scores was 6–30 where higher scores indicated more positive attitudes

*Informed choice defined as adequate knowledge and screening intentions aligned with screening attitudes

*SD* standard deviation

**Table 3:** Secondary Outcomes. The *p*-Value Provided Is for the Main Effect of Study Arm

	No. (%)			*p*-value
Variable	Control (*n* = 122)	Text (*n* = 132)	Animation (*n* = 122)	
Intention to speak with GP				.001
No	61 (50.0)	35 (26.5)	36 (29.5)	
Don’t know	16 (13.1)	24 (18.2)	23 (18.9)	
Yes	45 (36.9)	73 (55.3)	63 (51.6)	
Perceived risk of cancer				.111
Below average	29 (23.8)	31 (23.5)	40 (32.8)	
Average	80 (65.6)	91 (68.9)	78 (63.9)	
Above average	13 (10.7)	10 (7.6)	4 (3.3)	
Cancer worry				.659
Not worried at all	65 (53.3)	69 (52.3)	58 (47.5)	
A bit worried	39 (32.0)	38 (28.8)	47 (38.5)	
Quite worried	13 (10.7)	16 (12.1)	10 (8.2)	
Very worried	5 (4.1)	9 (6.8)	7 (5.7)	
Emotion	Mean (95% CI) *n* = 121	*n* = 131	*n* = 122	
Assured	5.53 (5.25–5.81)	5.17 (4.90–5.43)	5.34 (5.07–5.62)	.179
Relieved	5.65 (5.36–5.95)	5.08 (4.80–5.36)	5.17 (4.88–5.46)	.013
Informed	5.97 (5.74–6.20)	5.74 (5.52–5.96)	5.86 (5.63–6.09)	.377
Anxious	2.33 (2.02–2.65)	2.52 (2.22–2.81)	2.56 (2.25–2.87)	.564
Worried	2.38 (2.07–2.69)	2.38 (2.08–2.68)	2.53 (2.22–2.83)	.753
Confused	1.96 (1.69–2.23)	2.08 (1.82–2.34)	2.15 (1.88–2.42)	.612

Data are number of participants (%), unless otherwise stated. *CI* confidence interval, *GP* general practitioner

*N*s for emotion outcome differ due to missing data

### Primary Outcomes

#### Knowledge

Participants who received either intervention were significantly more likely to report adequate knowledge compared to controls (control 23.8%, reference; text 59.8%, RR = 1.90, 95%CI 1.51–2.39, *p* < 0.001; animation 68.9%, RR = 2.45, 95%CI 1.85–3.24, *p* < 0.001). There was no significant difference between intervention arms (RR = 1.29, 95%CI 0.92–1.80, *p* = 0.135). See Supplementary Table II for individual knowledge item results.

#### Screening Attitudes

Participants who received the video were significantly less likely to report high positive screening attitudes than controls (control 65.6%, reference; animation 40.2%, RR = 0.58, 95%CI 0.43–0.77, *p* < 0.001). There were no significant differences in screening attitudes between those who received the text information and controls (text 51.5%, RR = 0.71, 95%CI 0.53–0.96, *p* = 0.023) or between intervention arms (RR = 0.81, 95%CI 0.65–1.02, *p* = 0.070) with Bonferroni correction.

#### Screening Intention

Compared to controls, both intervention arms significantly reduced their intentions to screen (control 17.2%, reference; text 36.4%, RR = 1.30, 95%CI 1.12–1.52, *p* < 0.001; animation 49.2%, RR = 1.63, 95%CI 1.34–1.98, *p* < 0.001). There was no significant difference between intervention arms (RR = 1.25, 95%CI 1.01–1.56, *p* = 0.039).

#### Informed Choice (Composite Outcome)

Participants who received either the text or animation intervention were significantly more likely to make an informed choice compared to participants in the control arm (control 18.0%, reference; text 32.6%, relative risk [RR] = 1.22, 95% confidence interval [CI] 1.05–1.41, *p* = 0.008; animation 41.0%, RR = 1.39, 95%CI 1.17–1.65, *p* < 0.001). There was no significant difference between intervention arms (text vs. animation) (RR = 1.14, 95%CI 0.95–1.38, *p* = 0.165).

### Secondary Outcomes

Compared to controls, those who received either intervention were significantly more likely to intend to speak with their GP (control 36.9%, reference; text 55.3%, OR = 2.42, 95%CI 1.51–3.88, *p* < 0.001; animation 51.6%, OR = 2.09, 95%CI 1.30–3.37, *p* = 0.002). No significant differences were evident between textual information and animated video intervention arms in intention to speak with GP (OR = 0.87, 95%CI 0.54–1.39, *p* = 0.686). There was no difference between arms in perceived risk of cancer (*p* = 0.111) or cancer worry (*p* = 0.659).

Regarding emotional response to the BreastScreen letter, those who received the text (mean difference [MD] =  − 0.58, 95%CI =  − 0.98, − 0.17, *p* = 0.006) or animation (MD =  − 0.48, 95%CI =  − 0.89, − 0.07, *p* = 0.023) were less likely to report feeling relieved compared to control (mean = 5.66). There were no significant differences in the degree to which participants were assured, informed, anxious, worried, or confused in response to the letter across intervention or control arms (all *p* > 0.05).

### Sensitivity Analysis

All main effects for primary outcomes did not differ in a sensitivity analysis (*n* = 343) where all participants who reported being from Western Australia were removed (*n* = 33) (Supplementary Table III), as well as in analyses with adjusted thresholds for the primary outcomes (Supplementary Table IV).

## DISCUSSION

In this online randomized controlled trial, women aged 70–74 years who received information that explained the reasons for screening invitation cessation were more likely to make an informed choice about screening beyond 74 years than women who did not receive this messaging. Women who received the interventions had greater knowledge, less positive screening attitudes, and reduced screening intentions.

The animation intervention increased the proportion of participants making an informed choice by 24% compared to controls, and the text by 15%. Similarly, a breast cancer screening decision aid for women aged 70 years resulted in a 25% increase in informed choice compared to controls.^31^ Our findings have significance for implementation given that our developed interventions were brief, which would be relatively easy to scale-up across a population screening program compared to a decision aid which requires time and significant discussion for adequate development and use, presenting considerable challenges for implementation.^39^ Decision aids may still be useful and appropriate for older adults who desire more detailed information, as interventions at patient, clinician, and system levels are key to improving decision-making around screening for older people.^21,22^ Screening providers communicating direct to consumer may be a feasible and effective way to counter the negative beliefs women hold regarding the current recommendations for no longer being invited to screen.^18,19^ Given how challenging it is for consumers to grasp the concept of screening harms such as overdiagnosis,^40^ these findings also have wider implications for other screening programs, highlighting how overdiagnosis can be simply and effectively communicated. However, the context in which recommendations are communicated may have different impacts on how this messaging is received. For example, in the US, different levels of trust in the government-funded health insurance and controversy surrounding breast screening for older women may reduce the impact of this kind of messaging.

There are consequences of providing information that should also be considered. Firstly, our interventions led some older women to be more unsure about whether to continue or stop breast screening and more likely to want to speak with their GP. Clinicians may need support and training to navigate these discussions and provide recommendations if requested.^20,41^ Verbal scripts have been developed in the US that suggest how the idea of stopping cancer screening could be introduced, how a recommendation to stop could be explained with further reasoning, or how the benefits and harms of screening could be discussed in a shared decision-making conversation.^42^ These strategies for discussing stopping screening could be further adapted for use in contexts where national screening programs are implemented. Secondly, women who did not receive additional messaging reported greater relief in response to the BreastScreen letter. This may be explained by the relieving nature of receiving a letter from a trusted organization that provides a reassuring screen result without needing to consider additional information, or because information about screening benefits and harms reminds women that they are getting older and therefore feel a lack of control. Given women perceive mammograms as a way of looking after their health, this information could lead to feelings of distress.^43^

Health animations have shown promise in communicating complex information to people with low health literacy.^26^ There were no statistically significant differences observed between text and animation study arms. However, the format of information delivery is worth exploring in future studies with larger, more diverse samples. Animations may be particularly useful for low health literacy audiences when communicating conceptual knowledge rather than numerical risk information.^44^ Regardless, both intervention formats improved informed decision-making which could provide helpful flexibility in allowing for potential individual differences in needs and preferences for text or animation formats.

### Strengths and Limitations

This study was strengthened through randomization to study arms ensuring similar baseline characteristics of participants across arms. In addition, differences in outcomes can be attributable to the intervention. We also utilized survey software techniques to ensure quality responses (attention and consistency checks) and designed the messaging to potentially supplement existing BreastScreen communications and be easily implemented and scaled up.

There are limitations to this study. Recruitment through an online panel means that our sample is likely not representative of the women aged 70–74 years who would typically attend to BreastScreen services in Australia (i.e., underrepresentation of culturally and linguistically diverse and low socio-economic groups).^15^ Qualtrics randomly selects respondents but does not guarantee national representation. Our sample reported around 30% were not born in Australia, but approximately 40% of women attending BreastScreen are not born in Australia.^16^ Furthermore, this study was hypothetical in nature. Older women who receive this information may respond differently in real-life settings. Further research is needed, such as a clinical pilot trial conducted through BreastScreen services or other screening programs to understand the feasibility and impact of these interventions in practice, with more diverse samples.

We also cannot guarantee all participants paid attention to the entire intervention content despite restricting ability to proceed in the survey after a reasonable time. Additionally, there was a slight difference in the content provided in the two versions of the intervention. Pre-existing beliefs about why screening invitations stop were explicitly acknowledged in the animation version (i.e., government cost-saving, decreased risk), but not in the text version. Future research should test different elements of the information provided to understand which parts are more effective among different audience types, and whether directly addressing pre-existing beliefs is important in supporting informed choice.

Our interventions aimed to align with the BreastScreen Australia upper age limit for screening invitations (74 years), but we recognize there is no consensus on this matter. They were therefore limited in covering the nuanced information likely required for informed choice for some older women, but rather general information as a first step to be further elaborated with a primary care physician if desired. Further support is required for older women and clinicians navigating this decision. Another limitation is that intention rather than actual behavior was measured. However, lower screening intentions have been shown to be associated with lower screening rates.^45^

Finally, there are ongoing discussions around what thresholds should be set to define an informed choice.^29^ Our sensitivity analyses showed that the overall main effects remained significant with different thresholds. Using a threshold of below 30 on the attitudes scale as “less positive screening attitudes” due to the high within-sample median is justified in the context of this study. The public has well-documented strong positive attitudes toward cancer screening,^46,47^ which are particularly influential in older adults’ cancer screening decisions^48,49^ (likely due to a longer period of exposure to persuasive screening messaging). Scoring less than the maximum score on this scale may in fact have clinical significance.

## CONCLUSIONS

Messaging outlining the rationale for cessation of breast screening invitation to women aged 70–74 years in this online randomized controlled trial improved informed choice and knowledge, reduced positive screening attitudes, and lowered screening intentions. This study is an important first step in improving the messaging provided by national cancer screening programs direct to older adults and supporting more informed choices.

### Supplementary Information:

Below is the link to the electronic supplementary material.

Supplementary file1 (PDF 351 KB)

## Data Availability

Data are available upon reasonable request. De-identified participant data will be made available upon request to anyone wishing to access it who provides a methodologically sound proposal to the principal investigator. The contact detail of the principal investigator is kirsten.mccaffery@sydney.edu.au.
