# Supplementary material for: Randomized Trial of Information for Older Women About Cessation of Breast Cancer Screening Invitations
Source: J Gen Intern Med. 2024 Feb 26;39(8):1332–41. doi: 10.1007/s11606-024-08656-3 (PMC11169431; doi:10.1007/s11606-024-08656-3)
Supplement: Supplementary file 1 — Supplementary file1 (PDF 351 KB) [file 11606_2024_8656_MOESM1_ESM.pdf]

SUPPLEMENTARY MATERIAL

Supplementary Table I. Primary and secondary outcome measures

**Outcome**	**Measure**
**Primary outcomes**	
Informed choice	Composite measure: participants made an 'informed choice' if they reported adequate knowledge and screening intentions aligned with screening attitudes
Screening intention	Single item on 5-point scale: definitely will to definitely will not<br>'Do you intend to continue screening beyond 74 years?'<br>Dichotimised to 'intend to screen' (probably/definitely will) or 'intend not to screen' (unsure or probably/definitely will not)
Knowledge (conceptual)	Eleven purpose-built true/false questions to assess knowledge of information about breast screening<br>Q1: The benefit of getting a mammogram is finding and treating a breast cancer at an early stage that would otherwise have harmed a woman. (T)<br>Q2: Mammograms can result in finding and treating a breast cancer that would not have otherwise caused a woman any problems. (T)<br>Q3: Women aged 75 or older have a lower chance of getting breast cancer than younger women. (F)<br>Q4: All breast cancers found by a mammogram would eventually have shown up or caused problems in a woman's lifetime. (F)<br>Q5: Women aged 50-74 years are more likely to experience the downsides of breast screening than women aged 74 years or older. (F)<br>Q6: As a woman ages and develops other health issues, the chance of experiencing the downsides of screening begins to outweigh the potential benefits. (T)<br>Q7: Women will not receive reminder letters to screen after turning 75. This is because they have a reduced chance of developing breast cancer. (F)<br>Q8: Women will not receive reminder letters to screen after turning 75. This is because the government is saving costs. (F)<br>Q9: Women will not receive reminder letters to screen after turning 75. This is because the downsides of screening are likely to outweigh the benefits. (T)<br>Q10: If women wish to continue screening after turning 75, they will need to pay. (F)<br>Q11: Because women aged 75 or older are no longer invited to breast screening, seeing their GP about changes in their breasts is less important. (F)
Screening attitudes	Six items on 5-point scale: strongly disagree to strongly agree (range 6-30). 'For you, having breast screening is:'<br>Beneficial, harmful*, a good thing, a bad thing*, worthwhile, important<br>Dichotomised to 'positive screening attitudes' (highest possible score – 30) or 'less than positive screening attitudes' (<30)
**Secondary outcomes**	
Intention to speak with GP	Single item on 3-point scale: yes, no, don't know<br>'After receiving this letter from BreastScreen, would you plan to talk to your GP about it?'
Perceived risk of cancer	Single item on 3-point scale: below average, average, above average<br>'Compared to other women your age and ethnicity, what do you think is your chance of getting breast cancer in your lifetime?'

Cancer worry	Single item on 4-point scale: not worried at all, a bit worried, quite worried, very worried<br>'How worried would you be about developing breast cancer after receiving this [illegible]
Emotional response	Six items on 7-point scale: not at all to extremely<br>'How do you feel about the letter you received from BreastScreen?'<br>Assured, relieved, informed, anxious, worried, confused

*Reverse coded

INTERVENTIONS

<u>**Study arm 1 (Control)**</u>

Please read the information below and answer the questions that follow. Please note that you will be asked to <u>imagine</u> the following information is true. Please answer how you would feel or react if you were in this situation, to the best of your ability.

Please imagine that you have gone to a routine mammogram screening for breast cancer and received the following letter about your mammogram results.

Your mammogram (breast x-ray) result:
No breast cancer could be seen on your mammogram

Thank you for visiting BreastScreen for your mammogram.

No breast cancer was seen by the doctors who looked at your mammogram.

When should you next visit for breast screening?
You are eligible for a mammogram **every 2 years** through BreastScreen.

There is a small chance that an existing breast cancer may not be seen on a mammogram. New breast cancers can develop between screening visits. If you notice any changes in your breasts you should contact your GP immediately.

BreastScreen sends reminder letters to women aged 50 to 74 years. However, all women over the age of 40 can access screening with BreastScreen by calling to arrange an appointment.

Thank you for attending BreastScreen.

Yours sincerely

Study arm 2 (Intervention 1)

Please read the information below and answer the questions that follow. Please note that you will be asked to imagine the following information is true. Please answer how you would feel or react if you were in this situation, to the best of your ability.

Please imagine that you have gone to a routine mammogram screening for breast cancer and received the following letter about your mammogram results.

Your mammogram (breast x-ray) result:
No breast cancer could be seen on your mammogram

Thank you for visiting BreastScreen for your mammogram.

No breast cancer was seen by the doctors who looked at your mammogram.

When should you next visit for breast screening?
You are eligible for a mammogram **every 2 years** through BreastScreen.

There is a small chance that an existing breast cancer may not be seen on a mammogram. New breast cancers can develop between screening visits. If you notice any changes in your breasts you should contact your GP immediately.

BreastScreen sends reminder letters to women aged 50 to 74 years. However, all women over the age of 40 can access screening with BreastScreen by calling to arrange an appointment. **If you turn the page, you can find more information about why women aged over 75 years are no longer reminded to undertake breast screening**.

Thank you for attending BreastScreen.

Yours sincerely

After 75… why am I no longer reminded to have breast screening?

- Breast cancer screening programs aim to benefit women by finding breast cancer early and lowering the chance that women die of breast cancer.
- But there are also downsides to breast screening. Screening may find breast cancer that was not going to cause a woman any problems if she did not screen.
- Screening is no longer advised for **ALL** women aged 75 years and older. As a woman ages and develops other health issues, the chance of experiencing the downsides of screening begins to outweigh the chance of benefiting from screening.
- Regardless of your age and whether you decide to stop screening or not, see your GP if you notice any changes, lumps or pain in your breasts.
- Women aged 75 and older have a higher chance of getting breast cancer, but these cancers often grow slowly and do not need to be found.
- Although you won't receive reminder letters anymore, whether to continue or stop having free breast screening is up to you. If you are unsure, you could speak with your GP to help decide.

Readability score: grade 9.5

Study arm 3 (Intervention 2)

Please read the information below and answer the questions that follow. Please note that you will be asked to imagine the following information is true. Please answer how you would feel or react if you were in this situation, to the best of your ability.

Please imagine that you have gone to a routine mammogram screening for breast cancer and received the following letter about your mammogram results.

Your mammogram (breast x-ray) result:
No breast cancer could be seen on your mammogram

Thank you for visiting BreastScreen for your mammogram.

No breast cancer was seen by the doctors who looked at your mammogram.

When should you next visit for your breast screen?
You are eligible for a mammogram **every 2 years** through BreastScreen.

There is a small chance that an existing breast cancer may not be seen on a mammogram. New breast cancers can develop between screening visits. If you notice any changes in your breasts you should contact your GP immediately.

BreastScreen sends reminder letters to women aged 50 to 74 years. However, all women over the age of 40 can access screening with BreastScreen by calling to arrange an appointment. **You can find more information about why women aged over 75 years are no longer reminded to undertake breast screening in the video on the next page**.

Thank you for attending BreastScreen.

Yours sincerely

ANIMATION VIDEO SCRIPT

Breast cancer screening programs aim to benefit women by finding breast cancer early and lowering the chance that women die of breast cancer.

But there are also downsides to screening.

Screening may detect breast cancer that was not going to cause a woman any problems if she did not screen. This woman may then receive treatment that is not needed, which can cause unwanted side effects.

Harmless breast cancers can be detected and treated because they are very slow growing. Compared to women aged 50-74 years, women aged 75 or older have a greater chance of being diagnosed with harmless, slow-growing cancer.

Some people think breast screening is no longer recommended for older women because the government wants to save money, because they have a decreased risk of developing breast cancer, or because they are being given up on.

However, there is a different reason that breast screening is no longer advised for ALL women aged 75 and older.

As a woman ages and develops other health issues, the chance of experiencing the downsides of screening begins to outweigh the chance of benefiting from screening.

Regardless of your age and whether you decide to stop screening or not, see your GP if you notice any changes, lumps or pain in your breasts.

Women aged 75 or older have a higher chance of getting breast cancer, but these cancers often grow slowly and do not need to be found.

Although you won't receive reminder letters anymore, whether to continue or stop having free breast screening is up to you. If you are unsure, you could speak with your GP to help decide.

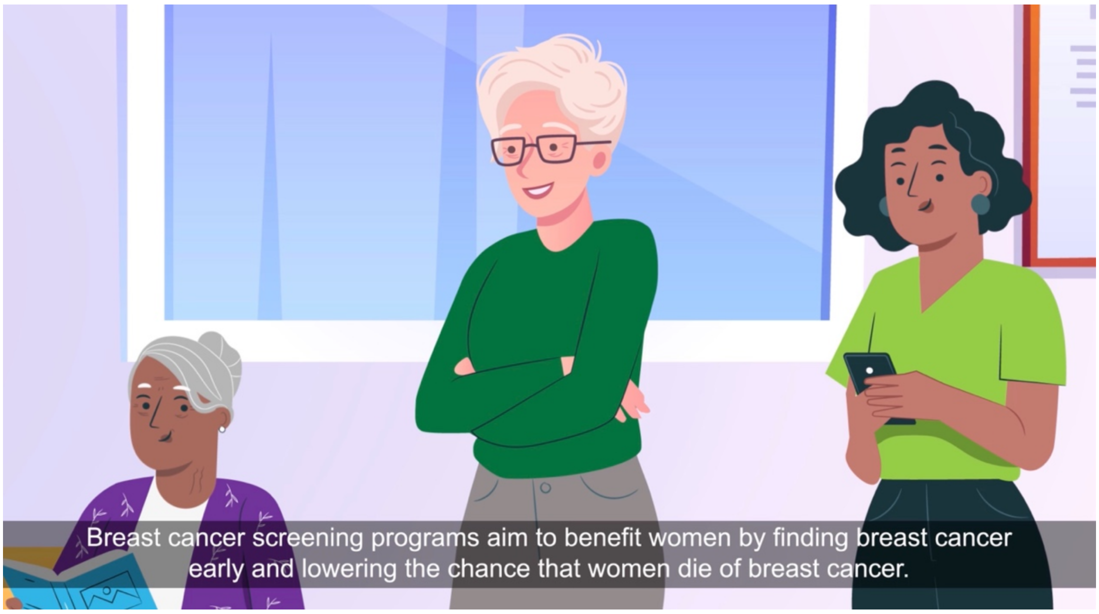

Evidence for information included in interventions:

Information	Evidence
This woman may then receive treatment that was not needed.	**Hersch, Jolyn, et al**. "Use of a decision aid including information on overdetection to support informed choice about breast cancer screening: a randomised controlled trial." *The Lancet* 385.9978 (2015): 1642-1652.
Harmless breast cancers can be detected and treated because they are very slow growing. Compared to women aged 50-74 years, women aged 75 or older have a greater chance of being diagnosed with a harmless, slow-growing cancer.	**Schonberg MA**, Marcantonio ER, Li D, Silliman RA, Ngo L, McCarthy EP. Breast cancer among the oldest old: tumor characteristics, treatment choices, and survival. *J Clin Oncol.* 2010;28(12):2038-2045.<br>**Diab SG**, Elledge RM, Clark GM. Tumor characteristics and clinical outcome of elderly women with breast cancer. *J Natl Cancer Inst.* 2000;92(7):550-556.
Some people think breast screening is no longer recommended for older women because the government wants to save money, because they have a decreased risk of developing breast cancer, or because they are being given up on.	**Smith J,** Dodd R, Wallis K, Naganathan V, Cvejic E, Jansen J, McCaffery K. Examining older adults' cancer screening decision-making: A qualitative interview study.
However, there is a different reason that breast screening is no longer advised for ALL women aged 75 and older.	**Jacklyn, G.,** et al. Impact of extending screening mammography to older women: Information to support informed choices. *International journal of cancer*. 2017; 141(8): 1540-1550.
Regardless of your age and whether you decide to stop screening or not, see your GP if you notice any changes, lumps or pain in your breasts.<br><br>Women aged 75 or older have a higher chance of getting breast cancer, but these cancers often grow slowly and do not need to be found.	**Schonberg MA**, Hamel MB, Davis RB, Griggs MC, Wee CC, Fagerlin A, Marcantonio ER. Development and evaluation of a decision aid on mammography screening for women 75 years and older. *JAMA internal medicine*. 2014 Mar 1;174(3):417-24.

Supplementary Table II. Knowledge individual items (% correct). The p-value provided is for the main effect of study arm.

**Knowledge individual item**	**Control (n=122)**	**Text (n=132)**	**Animation (n=122)**	**P value**
Benefit of getting mammogram is finding and treating breast cancer at early stage	124 (100.0)	129 (98.5)	125 (100.0)	.148
Mammograms can result in finding and treating breast cancer that would not have otherwise caused problems	106 (85.5)	122 (93.1)	119 (95.2)	.016
Women aged 75+ have lower chance of getting breast cancer than younger women	76 (61.3)	99 (75.6)	94 (75.2)	.018
All breast cancers found by mammogram would eventually have shown up or caused problems	41 (33.1)	60 (45.8)	68 (54.4)	.003
Women aged 50-74 years are more likely to experience downsides of breast screening than women aged 74+	76 (61.3)	81 (61.8)	82 (65.6)	.744
As a woman ages and develops other health issues, chance of experiencing downsides begins to outweigh benefits	39 (31.5)	102 (77.9)	108 (86.4)	<.001
Women will not receive reminder letters after turning 75 because…				
…they have a reduced chance of developing breast cancer	51 (41.1)	59 (45.0)	58 (46.4)	.685
…the government is saving costs	56 (45.2)	68 (51.9)	82 (65.6)	.004
…the downsides of screening are likely to outweigh the benefits	47 (37.9)	107 (81.7)	111 (88.8)	<.001
If women wish to continue screening after turning 75, they will need to pay	80 (64.5)	92 (70.2)	97 (77.6)	.075
Because women aged 75+ are no longer invited to screening, seeing their general practitioner about changes in their breasts is less important	115 (92.7)	117 (89.3)	104 (83.2)	.058

Supplementary Table III. Sensitivity analysis of primary outcome (women from Western Australia removed)

Variable	Control (n=109)	Text (n=120)	Animation (n=114)	P value
**Knowledge (total score dichotomised)**				
Adequate knowledge	25 (22.9)	74 (61.7)	78 (68.4)	<.001
**Total knowledge score (mean, SD, range 0-11)**	6.50 (1.26)	7.95 (1.74)	8.40 (1.93)	<.001
**Intentions about having breast screening**				
Intend to screen	90 (82.6)	76 (63.3)	56 (49.1)	<.001
Do not intend to screen	19 (17.4)	44 (36.7)	58 (50.9)	
Definitely will	63 (57.8)	44 (36.7)	26 (22.8)	…
Probably will	27 (24.8)	32 (26.7)	30 (26.3)	…
Unsure	11 (10.1)	27 (22.5)	30 (26.3)	…
Probably will not	5 (4.6)	12 (10.0)	24 (21.1)	…
Definitely will not	3 (2.8)	5 (4.2)	4 (3.5)	…
**Attitudes toward having breast screening[#]**				
Mean total attitudes score (SD)	28.60 (3.13)	27.18 (4.27)	26.54 (4.30)	<.001
**Attitudes (dichotomised)**				<.001
Most positive (score of 30)	73 (67.0)	62 (51.7)	46 (40.4)	
Less than positive (<30)	36 (33.0)	58 (48.3)	68 (59.6)	
**Informed choice* (composite outcome)**				
Made an informed choice	20 (18.3)	41 (34.2)	46 (40.4)	<.001

Data are number of participants (%), unless otherwise stated. [#]Attitude items were rated from strongly disagree (1) to strongly agree (5).
*Informed choice defined as adequate knowledge and screening intentions aligned with screening attitudes.
Range of possible scores were 6-30 where higher scores indicated more positive attitudes.
SD=standard deviation

Supplementary Table IV. Sensitivity analysis of primary outcome (adjusted thresholds for adequate knowledge, positive attitudes, and composite measure of informed choice)

Variable	Control (n=122)	Text (n=132)	Animation (n=122)	P value
**Knowledge (score range 0-11, dichotomized)**				
Adequate knowledge (main analysis; score ≥8/11)	*29 (23.8)*	*79 (59.8)*	*84 (68.9)*	*<.001*
Adequate knowledge (score ≥7)	65 (53.3)	106 (80.3)	99 (81.1)	<.001
Adequate knowledge (score ≥9)	7 (5.7)	49 (37.1)	65 (53.3)	<.001
**Attitudes (scale 6-30, dichotomized)[#]**				<.001
Positive (main analysis; score of 30)	*80 (65.6)*	*68 (51.5)*	*49 (40.2)*	*<.001*
Positive (score ≥24)	114 (93.4)	111 (84.1)	98 (80.3)	.010
**Informed choice* (composite outcome)**				
Made an informed choice (main analysis)	*22 (18.0)*	*43 (32.6)*	*50 (41.0)*	*<.001*
1: Knowledge score ≥7	60 (49.2)	83 (62.9)	81 (66.4)	.015
2: Knowledge score ≥9	6 (4.9)	34 (25.8)	55 (45.1)	<.001
3: Absolute attitudes value (score ≥24=positive)	22 (18.0)	48 (36.4)	51 (41.8)	<.001

Data are number of participants (%), unless otherwise stated.
[#]Attitude items were rated from strongly disagree (1) to strongly agree (5). Range of possible scores were 6-30 where higher scores indicated more positive attitudes.
*Informed choice defined as adequate knowledge and screening intentions aligned with screening attitudes.
